# A prospective multicenter study on prognostic factors and quality of care in Severe Acquired Brain Injury rehabilitation units: a project from the Tiresia network

**DOI:** 10.3389/fneur.2025.1527533

**Published:** 2025-02-19

**Authors:** Giovanni Nattino, Michele Acler, Stefano Bargellesi, Giannettore Bertagnoni, Greta Carrara, Antonio De Tanti, Anna Estraneo, Giulia Irene Ghilardi, Susanna Lavezzi, Francesco Lombardi, Lucia Francesca Lucca, Mauro Mancuso, Andrea Montis, Chiara Mulè, Jorge Navarro, Federico Posteraro, Elena Rossato, Laura Simoncini, Guido Bertolini

**Affiliations:** ^1^Istituto di Ricerche Farmacologiche Mario Negri IRCCS, Ranica, Italy; ^2^Ospedale Riabilitativo Villa Rosa, APSS Trento, Pergine Valsugana, Italy; ^3^AULSS3, Venezia, Italy; ^4^AULSS8 Berica, Vicenza, Italy; ^5^Centro Cardinal Ferrari, KOS Care, Fontanellato, Italy; ^6^IRCCS Fondazione Don Gnocchi, Firenze, Italy; ^7^Azienda Ospedaliero-Universitaria, Ferrara, Italy; ^8^Ospedale S. Sebastiano, Correggio, Italy; ^9^Istituto S. Anna, Crotone, Italy; ^10^Area Dipartimentale di Medicina Fisica e Riabilitativa, Azienda USL Toscana Sud Est; CRT - Clinica di Riabilitazione Toscana, Montevarchi, Italy; ^11^ASL Oristano, Oristano, Italy; ^12^Fondazione Poliambulanza, Brescia, Italy; ^13^Fondazione Don Gnocchi, Milan, Italy; ^14^USL Toscana Nord Ovest, Lucca, Italy; ^15^IRCCS Ospedale Sacro Cuore Don Calabria, Negrar di Valpolicella, Italy; ^16^Montecatone Rehabilitation Institute, Imola, Italy

**Keywords:** Severe Acquired Brain Injury, rehabilitation, prognostic factors, quality of care, multicenter study, Tiresia network

## Abstract

**Background:**

Severe Acquired Brain Injury (sABI) presents significant challenges in clinical management and rehabilitation due to its diverse and complex nature. The primary objective of the Tiresia project is to identify medium and medium-to-long-term prognostic factors for patients with sABI and to develop outcome indicators to evaluate the quality of care in rehabilitation units.

**Methods:**

This paper outlines the protocol for a prospective observational multicenter study conducted within the Tiresia Network. The study relies on a comprehensive data collection with stringent quality control measures. Ethical considerations emphasize patient privacy protection and adherence to regulatory standards. All of the Italian intensive rehabilitation units dedicated to sABI patients were eligible to participate in the study. Twenty-seven of them joined the project and started the data collection.

**Discussion:**

The present study represents a comprehensive effort to address critical gaps in sABI research and practice through a multicenter, prospective study design. Through rigorous data collection, analysis, and ethical oversight, the Tiresia project endorses the commitment of the research community to advancing evidence-based care and optimizing patient outcomes in sABI rehabilitation.

**Clinical trial registration:**

The study was registered on ClinicalTrials.gov (ID: NCT04905264), on 24 May 2021.

## 1 Introduction

Severe Acquired Brain Injury (sABI) is defined as a damage to the brain caused by an acute event. The origin can be traumatic and non-traumatic, such as vascular or hypoxic–ischemic. These events, which may result in temporary or permanent impairments, can profoundly affect motor, sensory, cognitive, emotional, and behavioral functions (1–3). The consequences of sABI are far-reaching, affecting not only the individuals directly impacted but also their families and broader social networks (4). The burden extends beyond the personal to include significant economic implications due to the costs associated with long-term care and rehabilitation (5, 6).

While the international literature offers insights into the global landscape of sABI (7, 8), unfortunately, there are no reliable statistics about its epidemiology in Italy. Nonetheless, extrapolations from data in other regions suggest that sABI is a major public health concern, with critical implications for the national healthcare system.

Effective management of sABI involves a continuum of care that spans from the acute phase, characterized by intensive resuscitative and neurosurgical interventions, to post-acute rehabilitation, aimed at optimizing functional recovery and quality of life (9). The complexity of sABI care is underscored by the diverse needs of patients across different phases of their recovery journey (10). Moreover, the absence of standardized approaches to rehabilitation and the heterogeneity in care delivery models across regions contribute to disparities in treatment outcomes and patient experiences (11).

Addressing the challenges inherent in sABI care requires a multifaceted approach that encompasses research, clinical practice, and policy initiatives. Central to this endeavor is the need to establish robust evidence regarding prognostic factors and quality of care in sABI rehabilitation. By elucidating the factors that influence patient outcomes and identifying best practices in care delivery, healthcare providers can optimize resource allocation, improve treatment efficacy, and enhance patient-centered care.

Tiresia, a network of Italian rehabilitation units dedicated to clinical research in rehabilitation medicine, represents a collaborative effort to advance knowledge and practice in sABI care (12). Through independent research projects and collaborative initiatives, Tiresia seeks to evaluate and improve the quality of care, promote evidence-based practices, and facilitate resource optimization in sABI rehabilitation.

This work presents the protocol of the first study of the Tiresia network, a prospective multicenter observational study aimed at establishing a robust framework to evaluate the quality of care in sABI units (13). Specifically, the study applies the well-established Donabedian’s model to evaluate the quality of healthcare based on outcome indicators (14).

## 2 Methods and analysis

### 2.1 Objectives

The overarching goal of the study is to establish a framework to evaluate the quality of care in sABI units based on outcome indicators. Using Donabedian’s model (14), an overall assessment of the quality of care of sABI units can be inferred from observed differences in patients’ outcomes after adjusting for differences, with respect to relevant prognostic factors, in the case-mix of the units. This project will therefore focus on the two key elements required in such a framework, i.e., the definition of the prognostic factors for sABI patients and the development of the outcome indicators to measure the quality of care.

Specifically, the primary objective of the Tiresia project is to identify medium and medium-to-long-term prognostic factors for sABI patients and evaluate their impact on rehabilitation outcomes. The study plans to identify key prognostic factors associated with each outcome measure at two different timings, 4 and 12 months after the injury.

The secondary objective of the study is to develop indicators of the quality of care provided by the participating rehabilitation units. In particular, the quality-of-care indicators will be based on the patients’ outcomes and span the multidimensional nature of the recovery assessment.

To formalize the research question using a standardized approach, we applied the Population, Intervention, Comparison, Outcome (PICO) framework (15). Specifically, the target population (P) includes all patients admitted to sABI units. The exposure of interest (or intervention, I) is the admission to each specific sABI unit, as the goal of the study is to compare the quality of care of each center to the average care, and there is no comparison (C). The primary outcomes (O), described in detail in Section 2.2, are impairments in body functions that are critical for functional independence.

### 2.2 Outcomes

Unlike traditional prognostic assessments, which often focus on mortality, this study primarily investigates the likelihood of functional recovery following the acute event. In particular, the study adopts a multidimensional assessment of the recovery.

The primary outcomes of the study are three functional outcomes related to critical events in the rehabilitation pathway: successful removal of the tracheostomy cannula, achievement of trunk control, and complete oral feeding resumption. Using the International Classification of Functioning, Disability and Health (ICF) to characterize impairments through an international standard for functioning and disability (16), the three outcomes are related to impairments in body functions and, specifically, to respiration functions (code b440), the tone of the muscles of the trunk (code b7355) and digestive functions (code b515). These outcomes are relevant to characterize the recovery of patients because they are critical markers of functional independence, as success in each of these tasks results in a drastic reduction in the patient’s need for assistance.

Three standardized and validated scales to investigate cognitive impairment, disability and autonomy are the secondary outcomes of the study: the Oxford Cognitive Screen (OCS) (17), the Glasgow Outcome Scale – Extended (GOS-E) (18) and the Supervision Rating Scale (SRS) (19), respectively.

Notably, all of the identified outcome measures are strategic to evaluate the quality of the provided care because they require the capacity to effectively organize the work of a broad interdisciplinary staff, including physicians, nurses, physiotherapists, psychologists, speech and language therapists, and occupational therapists.

### 2.3 Study design

The study adopts an observational, multicenter, prospective design. The multicenter nature of the study, which is facilitated by the existing Tiresia network, allows for the recruitment of a diverse patient population, enhancing the generalizability of study findings and facilitating collaborative research efforts.

### 2.4 Participating centers and study population

All Italian intensive rehabilitation units dedicated to sABI patients are eligible and invited to participate in the study. This inclusive approach ensures representation from heterogeneous clinical settings and geographic regions, enriching the study’s external validity and enabling the exploration of regional variations in care delivery and outcomes. A total of 27 centers participated in the study.

All adult patients with sABI consecutively admitted to the participating units during the study period are eligible for the study. Patients admitted later than 14 weeks after the acute event are excluded as, for these patients, evaluating the prognostic role of risk factors at the admission to the rehabilitation unit and the achievement of the outcomes of interest within the stay in the unit would be pointless, due to the closeness of the potential study enrollment and the first timing of outcome evaluation (i.e., 4 months).

### 2.5 Recruitment

The study duration spans 4 years, encompassing patients’ enrollment, follow-up assessments, and data analysis. We planned to enroll new patients in the study for 30 months. An additional 12-month period will be necessary to collect data on the 12-month follow-up for the last patients enrolled in the study.

### 2.6 Data collection

Central to the study design is the comprehensive collection of patient characteristics and rehabilitation outcomes, facilitated through the development of an ad-hoc electronic Case Report Form (eCRF), which serves as the primary tool to ensure the uniformity and accuracy of data across participating centers. The data collection is organized in three forms corresponding to different timepoints: sABI admission, 4-month and 12-month assessments. The first form describes the conditions of the patient at admission to the sABI unit and includes demographics, socioeconomic factors (e.g., working status, education level, caregiver support), comorbidities, characteristics of the acute event (e.g., timing, etiology), interventions and procedures received in the acute phase (e.g., intubation, tracheostomy), procedures undergoing at sABI unit admission (e.g., mechanical ventilation, O_2_ therapy), colonizations and infections by Multi-Drug Resistant Organisms (MDRO), parameters describing the conditions at sABI unit admission (e.g., heart rate, respiratory rate) and scores (e.g., Level of Cognitive Functioning – LCF, Early Rehabilitation Barthel Index – ERBI, Disability Rating Scale – DRS). At the 4- and 12-month follow-up assessments, the staff of the participating centers is required to evaluate the outcome measures of interest for the study, either through an on-site visit or a telephonic/video conferencing interview. Furthermore, upon registration for the study, sABI units are required to fill out a form describing their structural and organizational characteristics.

To ensure data quality and integrity, the study implements robust quality control measures at multiple levels. Each severe sABI Rehabilitation Unit designates an experienced clinician responsible for protocol adherence and data integrity at the local level. The Coordination Center holds regular meetings with representatives from the participating centers to address any challenge with the study, provide guidance, and ensure consistency and standardization in the collected data across all units. An indexed operating manual describing the data collection items has been developed and is accessible through a simple click during data entry. In addition, the detailed definition of the variables to be collected is clearly displayed on the screen, to avoid misinterpretation of the required fields. A real-time set of controls operates during data collection and includes completeness checks, warnings and errors to promptly highlight discrepancies in the inputted information. The rules for defining warnings and errors will be continuously revised and implemented based on user suggestions. They will be of five types: validity, plausibility, logical congruence, clinical congruence, and definition congruence. Furthermore, data will be centrally processed for epidemiological inconsistencies that cannot be automatically detected during data entry.

### 2.7 Statistical analysis

The statistical analysis of study data is conducted by the Tiresia Coordination Center at the Istituto di Ricerche Farmacologiche Mario Negri IRCCS. Analyses will employ various analytical techniques to address the study objectives and research questions. Descriptive statistics, including proportions, medians and interquartile ranges, and means and standard deviations, will be used to summarize and present the collected data.

The primary analysis focuses on identifying prognostic factors predictive of the identified outcome measures and in particular: the achievement of the three objectives in the rehabilitation process (i.e., successful decannulation, trunk control and complete oral feeding); a pathological result in the five cognitive domains investigated by the OCS (attention, language, numerical cognition, praxis and memory) (20); the value of the GOS-E scale, simplified into 4 levels (death or vegetative state, severe disability, moderate disability, good recovery); the value of the SRS scale, simplified into five levels (independent, night supervision, part-time supervision, indirect full-time supervision, direct full-time supervision). Separate regression models will be developed for each outcome and timing (4 and 12 months from the acute event). Logistic regression models will be used to assess the achievement of the rehabilitation objectives and the abnormalities in the OCS cognitive domains, which are dichotomous variables. Multinomial logistic regression models will be used to handle the GOS-E and SRS scales, which are categorical variables with four and five levels, respectively. To account for the large number of candidate predictors to screen as potential prognostic factors, we will use the technique of Least Absolute Shrinkage and Selection Operator (LASSO) (21), which is a popular approach to perform variable selection when the number of potential predictors is large compared with the sample size (22).

The secondary analysis of the study is aimed at developing quality of care indicators for sABI Units. This step will leverage the prognostic models developed for the main analysis, which will be employed to calculate the expected number of failures for each dichotomous outcome and the expected value of each of the possible levels for the categorical variables. Because the models will be developed using all patients admitted to all participating centers, they represent the average behavior and can be used as a reference (or benchmark) for quality-of-care assessment. The comparison in each sABI Unit between the expected and observed frequencies of the outcomes will constitute the indicator of quality of care for the department. The most common approach to perform this comparison is the standardized event ratio (SER), i.e., the ratio of observed and expected events (23). A SER value less than 1 indicates a lower-than-average number of events, while a value greater than 1 indicates that a center experienced more events than expected. Each SER will be provided with the corresponding confidence interval. Importantly, SER heavily relies on the prognostic model used as a benchmark of quality of care. In order to assess the robustness of the results against the prognostic model developed, indicators based on nonparametric methods, independent of that model, will also be used. In particular, a matching-based design will be applied (24). For each center, each patient will be matched to *K* other patients admitted to different units but similar to the selected patient with respect to the identified prognostic factors. Matching will be performed using state-of-the-art algorithms, including optimal and cardinality matching. This strategy will create a control group for each center with a similar case mix with respect to the relevant prognostic factors. The quality of care will be measured in terms of risk difference for each failure indicator, provided with the appropriate confidence interval. The parameter *K*, which sets how many control subjects will be matched to each patient, will be chosen based on the size of the database. We expect to be able to perform this analysis with a value of *K* between 1 and 3.

### 2.8 Sample size calculation

Each sABI Unit admits an average of 60 patients per year. When the study was designed, we assumed that 15 centers would have actively enrolled patients. Accordingly, we expected to collect data on about 900 patients per year. To develop a logistic regression model, it is common to consider adequate a sample that has at least 10 events for each predictor included in the model (25). Extending the data collection for 30 months and assuming that failure indicators are infrequent (10% of the case series), it will be possible to develop predictive models that include 10% * 2,250/10 = 22 predictors. Thus, we will be able to investigate the independent prognostic value of several factors and, with such a large number of predictors, it will be possible to develop accurate prognostic models to be used in the planned quality-of-care indicators.

## 3 Study monitoring

The Tiresia Coordination Center oversees the centralized monitoring of study data, ensuring adherence to study protocols, data quality standards, and ethical guidelines. Through regular quality reports and ongoing communication with participating centers, the Coordination Center will identify and promptly address data discrepancies, protocol deviations, and emerging issues. Sample-based site visits, including virtual meetings and on-site assessments, further enhance data monitoring and quality assurance efforts, fostering collaboration and accountability among study stakeholders.

## 4 Ethics and dissemination

Ethical approval for the study has been granted by the Section of the Lombardy Region of Fondazione Don Gnocchi IRCCS Ethics Committee (study code: 03_07/04/2021). The study adheres to stringent ethical standards and regulatory guidelines to protect patient privacy and the confidentiality of the collected information throughout the research process. Informed consent procedures ensure that patients or their legal representatives are fully informed about the study objectives, procedures, and potential risks before providing consent for participation. Special provisions are made for patients unable to provide informed consent due to the severity of their clinical condition, ensuring that their rights and interests are safeguarded in accordance with ethical guidelines and regulatory requirements. In Italy, whenever the patient does not have a legally recognized representative and has never been, while in the rehabilitation unit, in the conditions to give truly informed consent, consent can be waived or delayed. In particular, at the moment when the ethical approval was granted, the Italian Data Protection Authority (DPA) provided special prescriptions for the processing of data that are necessary for the conduct of studies enrolling persons who, due to the severity of their clinical condition, are unable to understand the indications made in the information and validly give consent (26). The DPA authorized the processing of personal data of such patients, even in the absence of their informed consent, subject to the limits and conditions specified in the provision (Chapter 5 of Annex 1: “Prescrizioni relative al trattamento dei dati personali effettuato per scopi di ricerca scientifica”). Specifically, these conditions are met in the specific case of patients without a legally recognized representative who are not in the condition of giving informed consent. Relatives or persons closest to the patient will be informed of the research in any case.

All requirements about communication, dissemination and security indicated by the DPA for the processing of sensitive data will be respected. In particular, data security measures are implemented to protect sensitive patient information and comply with national and European regulations governing the use of personal data for research purposes. Patient data are pseudonymized and encrypted to prevent unauthorized access or disclosure, with access restricted to authorized personnel via secure authentication mechanisms. Data storage and transmission protocols adhere to strict security standards, minimizing the risk of data breaches or unauthorized access.

## 5 Conclusion

The present study, promoted by the Tiresia network, represents a comprehensive effort to address critical gaps in sABI research and practice through a multicenter, prospective study design. Specifically, the overarching goal of the study is to assess the quality of care in sABI units using outcome indicators while accounting for differences in patient case-mix based on prognostic factors measured at admission. Notably, the study is not focused on the assessment of the effectiveness of specific treatments, interventions or policies. Determining treatments associated to the best outcomes is a medium-to-long term goal of the Tiresia network, which can be achieved by performing audits and further investigations in the centers showing a quality of care significantly deviating from the average. This study will therefore offer the possibility to generate hypotheses on the effectiveness of methods of care, which will be tested with further studies, possibly extending the data collection and leveraging the formed network of sABI units.

A distinctive and innovative feature of the study is the unprecedented number of participating centers (27 sABI units), whose involvement has been facilitated by the Tiresia network. The study aims at establishing one of the largest data collections in the field in Italy and internationally in terms of patient enrollment. Such a broad participation provides a robust platform for collecting diverse and comprehensive data, enhancing the generalizability and relevance of study findings.

Another strength of the study lies in its robust underlying methodology, which is evident in several aspects of the study design. These include the standardized definition of primary outcome measures using the ICF framework, the implementation of stringent data quality control procedures to ensure consistency in data collection across sites, and the application of advanced statistical methods to identify deviations from the average quality of care among the centers.

This research endeavor will be a unique opportunity to advance knowledge and improve outcomes for sABI patients across Italy and beyond by elucidating prognostic factors and developing quality-of-care indicators. Through rigorous data collection, analysis, and ethical oversight, the Tiresia project endorses the commitment of the research community to advancing evidence-based care and optimizing patient outcomes in sABI rehabilitation.
